# Azurin a potent anticancer and antimicrobial agent isolated from a novel *Pseudomonas aeruginosa* strain

**DOI:** 10.1038/s41598-025-86649-w

**Published:** 2025-01-30

**Authors:** Nourhan A. Zaghloul, Mona K. Gouda, Yasser Elbahloul, Nancy M. El Halfawy

**Affiliations:** Department of Botany and Microbiology, Faculty of Science, Alexandria University, Alexandria, Egypt

**Keywords:** Azurin, *Pseudomonas aeruginosa*, Anticancer, Antimicrobial, Blue-copper protein, Antimicrobials, Applied microbiology, Microbiology

## Abstract

Azurin, a bacterial blue-copper protein, has garnered significant attention as a potential anticancer drug in recent years. Among twenty *Pseudomonas aeruginosa* isolates, we identified one isolate that demonstrated potent and remarkable azurin synthesis using the VITEK 2 system and 16S rRNA sequencing. The presence of the azurin gene was confirmed in the genomic DNA using specific oligonucleotide primers, and azurin expression was also detected in the synthesized cDNA, which revealed that the azurin expression is active. Furthermore, crude azurin protein was extracted, precipitated using 70% ammonium sulfate, dialyzed, and subjected to purification using carboxymethyl-Sephadex in affinity chromatography as a cheap method for purification. The partially purified azurin protein was characterized using polyacrylamide gel electrophoresis, energy-dispersive X-ray spectroscopy, Fourier-transform infrared spectroscopy, and nuclear magnetic resonance spectroscopy. Notably, qualitative elemental analysis by EDX showed the presence of copper and sulfur, corresponding to the copper-core and disulfide-bridge, respectively, in the purified azurin fraction. Moreover, FTIR spectroscopy revealed characteristic amide I and II absorption peaks (1500–1700 cm^− 1^), revealing the possible secondary structure of azurin. The results of NMR revealed the presence of characteristic amino acids such as methionine and cysteine, which confirmed the EDX results for sulfur-containing amino acids. Purified azurin exhibited antimicrobial activity against *Staphylococcus aureus*, *Bacillus subtilis*, *Escherichia coli*, and *Klebsiella pneumoniae*. Additionally, its anticancer properties were determined using the MTT assay and cell cycle analysis, revealing a preference for inhibiting the MCF7 breast cancer cell line where breast cancer is most common in Egypt. Overall, the research findings suggest that the local isolate, *P. aeruginosa* strain 105, could be a potential source of azurin protein for incorporation into cancer treatment strategies.

**Supplementary Information:**

The online version contains supplementary material available at 10.1038/s41598-025-86649-w.

## Introduction

Cancer poses a significant threat to human health, diminishing quality of life and ultimately leading to patient mortality. Traditional chemotherapeutic drugs often come with high toxicity and do not distinguish between cancerous and healthy cells, frequently resulting in severe side effects^1^. Breast cancer is the most common cancer among Egyptian women, representing 34.9% of total cancer cases and the leading cause of high rates of mortalities^2^. Globally, 2.3 million women were diagnosed with breast cancer and 670,000 deaths in 2022^3^. Most women who develop breast cancer in a high-income country will survive; the opposite is true for women in most low-income and middle-income countries^4^. This necessitates the development of novel, more efficacious anticancer drugs with minimal adverse effects, aiming for an optimal therapeutic outcome^5,6^. Emerging chemotherapeutic approaches include utilizing bacteria and their metabolites for cancer treatment^7,8^. Potentially therapeutic bacterial peptides offer several advantages. They are small in size, exhibit high specificity and affinity for inhibiting various cancer cell lines, readily penetrate cell membranes, and exhibit minimal drug-drug interactions^9–11^. For instance, bacteriocins, ribosomally synthesized antimicrobial peptides, are promising in cancer treatment as they can inhibit tumor development through mechanisms such as inducing apoptosis, blocking the cell cycle, and inhibiting cell metastasis^12^. Furthermore, the proteinaceous secretory metabolites of probiotic bacteria, such as *Enterococcus hirae*, were used as antiproliferative agents against various human cancer cell lines in a dose-dependent manner^13^. Moreover, *Bifidobacterium* and *Lactobacillus* spp. revealed anticancer properties in preclinical studies by several mechanisms, including inducing cancer cell apoptosis, inactivating carcinogenic toxins, and modulating host immunity^14^.

Azurin, a high-affinity copper-binding periplasmic protein that is known to be secreted in the growth medium at the late growth stage of *P. aeruginosa*, functions as a secondary metabolite and electron transport cofactor^6,15,16^. Azurin shows promise as a source of therapeutic peptides due to its reported anticancer, antiparasitic, and anti-HIV properties^17^. Its structure includes an extended α-helix with protein transduction activity, a C-terminal four-loop region structurally similar to immunoglobulin variable domains, and a β-sandwich core with an immunoglobulin fold, which may contribute to its ability to evade the immune response and exert its anticancer effects^6,18,19^. Notably, a functional 28-amino acid fragment of azurin, designated p28, has been approved by the Food and Drug Administration (FDA) as a safe and effective anticancer treatment for both adult and pediatric patients^6^. Clinically, p28 as a single therapeutic agent was tested in two phase I clinical trials and granted the FDA Orphan Drug and Rare Pediatric Disease designations as it demonstrated preliminary efficacy without apparent adverse effects, toxicity, or immunogenicity in pediatric patients with recurrent and refractory central nervous system tumors and in adult patients with advanced solid tumors^20–22^.

Studies have demonstrated that azurin possesses promising anticancer therapeutic properties, particularly against breast cancer, by interfering with various signaling pathways^6,23^. This selectivity stems from azurin’s ability to penetrate cancer cells, inducing cytotoxicity and programmed cell death (apoptosis) while remaining non-toxic to normal cells^24,25^. Furthermore, azurin exhibits a strong affinity for the tumor suppressor protein p53, a key regulator of cell growth, death, and genomic stability^26^. Azurin also targets the Ephrin type-B receptor 2-mediated (EphB2) cell proliferation pathway, a signaling cascade involving a family of overexpressed tyrosine kinase receptors in many tumors^27^.

This study aims to isolate a novel azurin-producing *P. aeruginosa* strain from an Egyptian habitat. The isolated strain will undergo characterization, focusing on its in vitro anticancer activity against the MCF7 breast cancer cell line, particularly its apoptotic induction potential. Furthermore, the strain’s antimicrobial activity will be evaluated.

## Results

### Screening and identification of azurin-producing bacteria

**Fig. 1:**
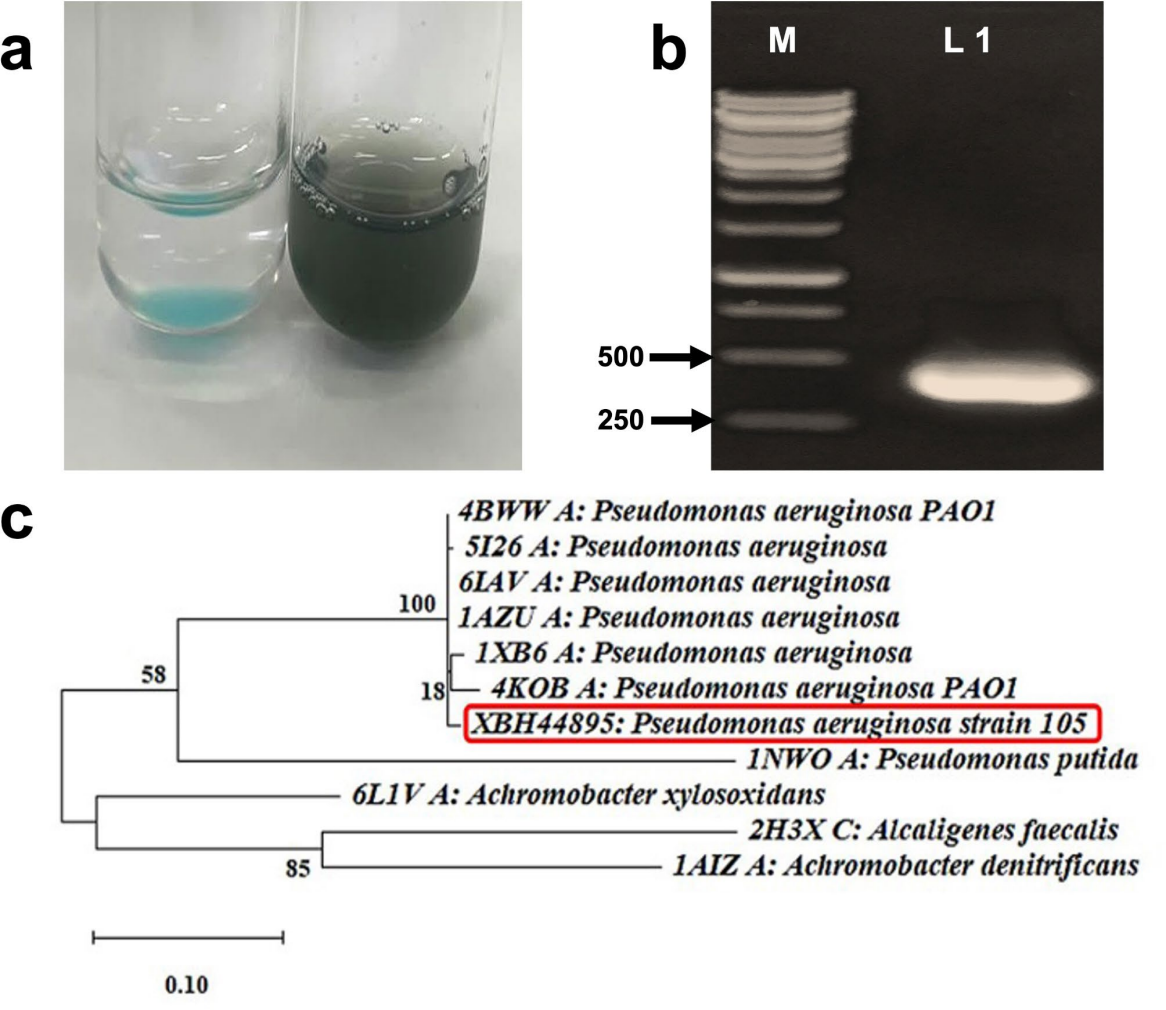
Detection of azurin in isolate 105. **(a)** CuCl_2_ test. Lane 1: Negative control. Lane 2: Periplasmic extract from isolate 105 showing a dark blue color indicative of azurin production after 24 h of incubation. **(b)** Agarose gel electrophoresis for detection of the azurin gene. Lane M: 1 kb Gene Ruler (Fermentas, UK), Lane 1: PCR amplicon of the azurin gene (*azu*) from isolate 105 with the expected size of 442 bp. **(c)** Maximum Likelihood phylogenetic tree of *Pseudomonas aeruginosa* strain 105 based on azurin protein sequence.

Colonies on the isolation plates were visually inspected, and only those exhibiting a greenish color were selected as potential *Pseudomonas* sp. candidates for azurin production (Supplementary Table 1). One isolate, designated as strain 105, was chosen for further analysis based on its ability to produce azurin, as confirmed by measuring the absorbance ratio (Abs_625_/Abs_280_) of periplasmic extract using UV-Vis spectrophotometer and the range for azurin was about 0.54. The CuCl₂ test was performed on the periplasmic extract, and the development of dark blue color is indicative of azurin production (Fig. 1a). In addition, the presence of the azurin gene in the isolate’s genomic DNA was verified using PCR (Fig. 1b; Supplementary Fig. S1), and the amplicon sequencing revealed 99.65% similarity with the published azurin. The gene encoding azurin was deposited in the GenBank database under accession number PP848219. Otherwise, the physicochemical properties of the amino acid sequence revealed a protein with a theoretical pI of 6.39, an aliphatic index of 84.32, and a grand average of hydropathicity (GRAVY) of -0.099. Furthermore, the maximum likelihood phylogenetic relationship between the translated sequence of azurin protein from strain 105 and other related strains available in the NCBI database is represented in Fig. 1c. The results revealed that azurin from strain 105 is closely related to *P. aeruginosa* PAO1 azurin protein. Additionally, conventional PCR confirmed the expression of the azurin gene in the synthesized cDNA. Subsequent identification using the VITEK 2 GN ID card revealed the isolate to be *P. aeruginosa* with a 97% probability (Supplementary Table 2).

### Molecular identification and phylogenetic analysis using 16S rRNA

**Fig. 2:**
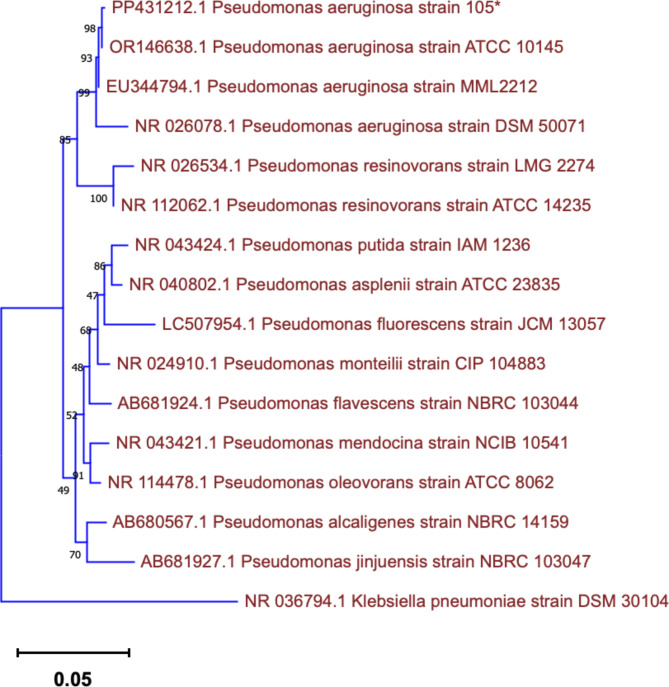
Neighborhood-joining tree based on 16S rRNA sequences showing the phylogenetic relationships between *Pseudomonas aeruginosa* strain 105 and reference strains present in the NCBI database. The tree was constructed with MEGA11.0, and the genetic distances were computed using Kimura’s 2-parameter model. The bootstrap value of 1000 replicates and the scale bar indicates a genetic distance of 0.05.

The 16S rRNA gene sequence of isolate 105 was amplified and sequenced for species-level identification. The obtained sequence was compared against the National Biotechnology Information Centre (NCBI) database using the Basic Local Alignment Search Tool (BLAST) to identify the closest relatives. The BLAST results confirmed the isolate as *P. aeruginosa*. For further characterization, the 16S rRNA gene sequence was aligned with reference sequences retrieved from NCBI using CLUSTALW software. Pairwise genetic distances were calculated using Kimura’s 2-parameter model. A phylogenetic tree was then constructed using these distances with a bootstrap value of 1000 replications (Fig. 2). The 16S rRNA-encoding gene could be reached in the GenBank database under accession number **PP431212**.

### Growth of *P. aeruginosa* 105 and azurin production

**Fig. 3:**
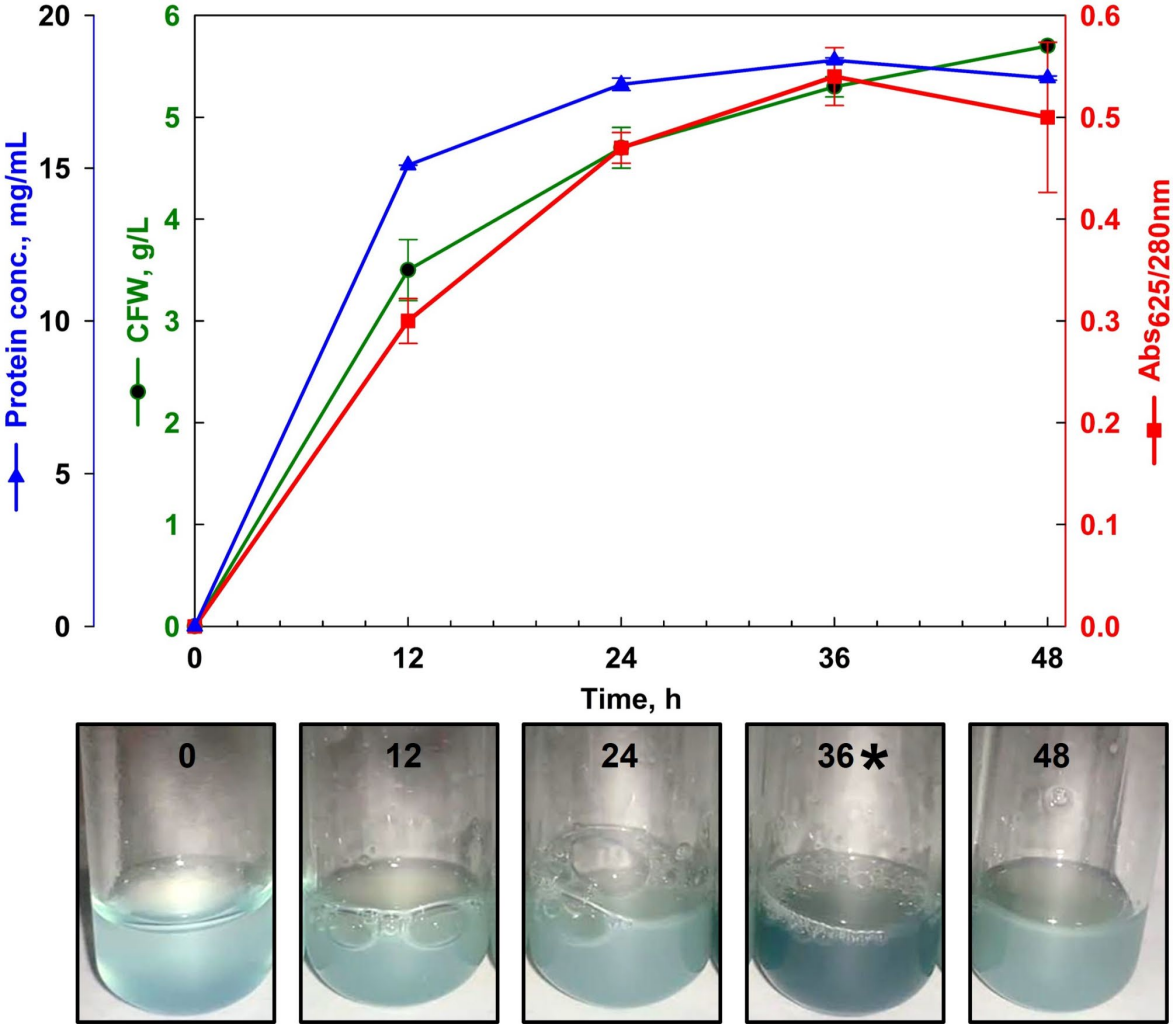
Azurin production by *Pseudomonas aeruginosa* strain 105. **(a)** Growth and azurin production over time. Cells were cultured in a production medium at 150 rpm and 32 °C. Azurin concentration was measured using the Bradford assay and the A_625_/A_280_ ratio. Data represent the mean ± SD of two independent experiments. **(b)** Azurin detection using the CuCl₂ method.

The growth profile and azurin production of *P. aeruginosa* strain 105 were investigated. To determine the optimal period for azurin production, the strain was aerobically cultured in 100 mL of production medium broth. Protein and azurin concentrations in the cell lysate were periodically monitored using three methods: the Bradford assay, the A_625_/A_280_ ratio, and the CuCl_2_ method **(**Fig. 3**)**. The results demonstrated a concurrent increase in protein and azurin concentrations. The highest azurin production was observed after 36 h, coinciding with an A_625_/A_280_ ratio of approximately 0.54.

### Purification of azurin

**Fig. 4:**
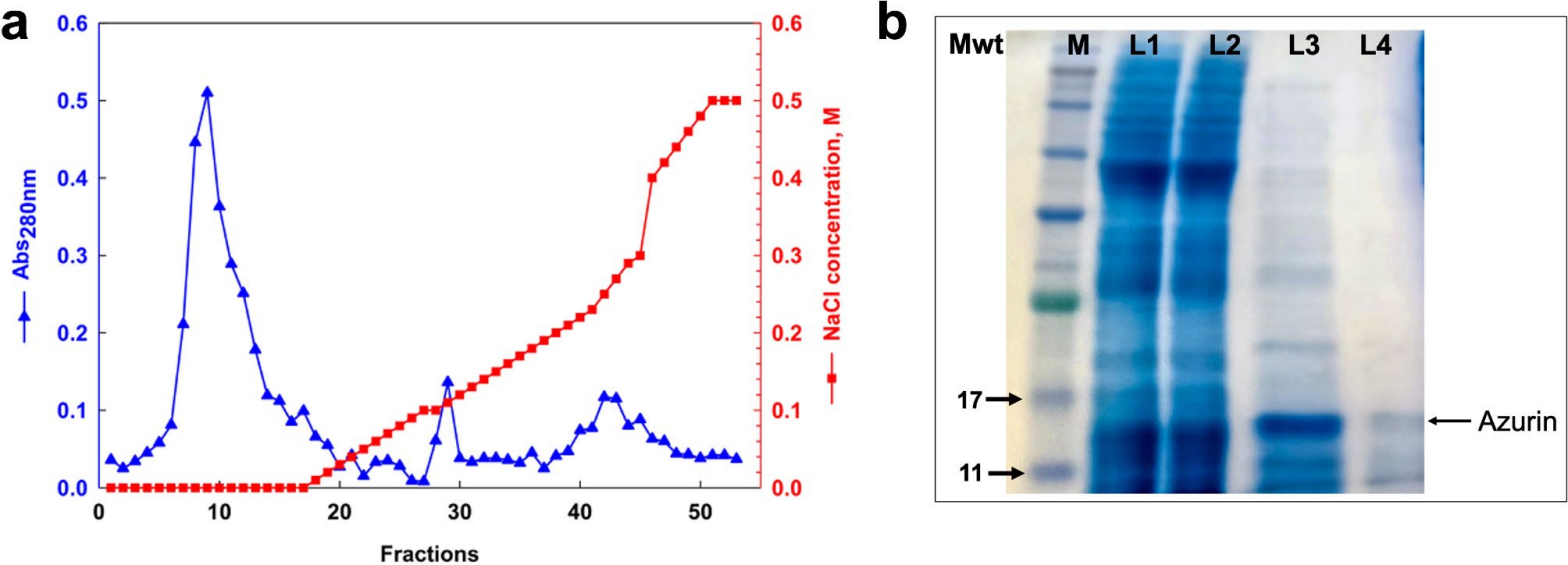
**(a)** Elution profile of azurin using CM-Sephadex column chromatography through the purification process. The column was washed with phosphate citrate buffer (pH 6.0) and eluted with NaCl solution (0, 0.2, 0.3, and 0.5 M) in the same buffer at a flow rate of 1.0 mL/min. The presence of proteins in eluents was measured with UV-Vis at Abs_280_. **(b)** SDS-PAGE of azurin fractions. Lane M: protein marker (Maestrogen, China), Lane 1: ammonium precipitated protein, Lane 2: concentrated protein with a 30 kDa MWCO membrane, Lane 3: fraction 1 semi-purified azurin protein, Lane 4: purified azurin protein.

Cation-exchange chromatography using CM-Sephadex revealed three distinct fractions (F1, F2, and F3) following elution **(**Fig. 4a**)**. Protein concentration in each fraction was quantified by measuring absorbance at 280 nm. SDS-PAGE analysis of the fractions indicated that F1 (Lane 3) was partially purified; thus, the fraction was reloaded on the CM-Sephadex column to obtain a purified protein. The purified azurin fraction (Lane 4) revealed a protein band with a corresponding molecular weight of approximately 14 kDa **(**Fig. 4b**)**. This fraction (F1) was subsequently freeze-dried and subjected to further physicochemical characterization.

### FTIR analysis of azurin structure

**Fig. 5:**
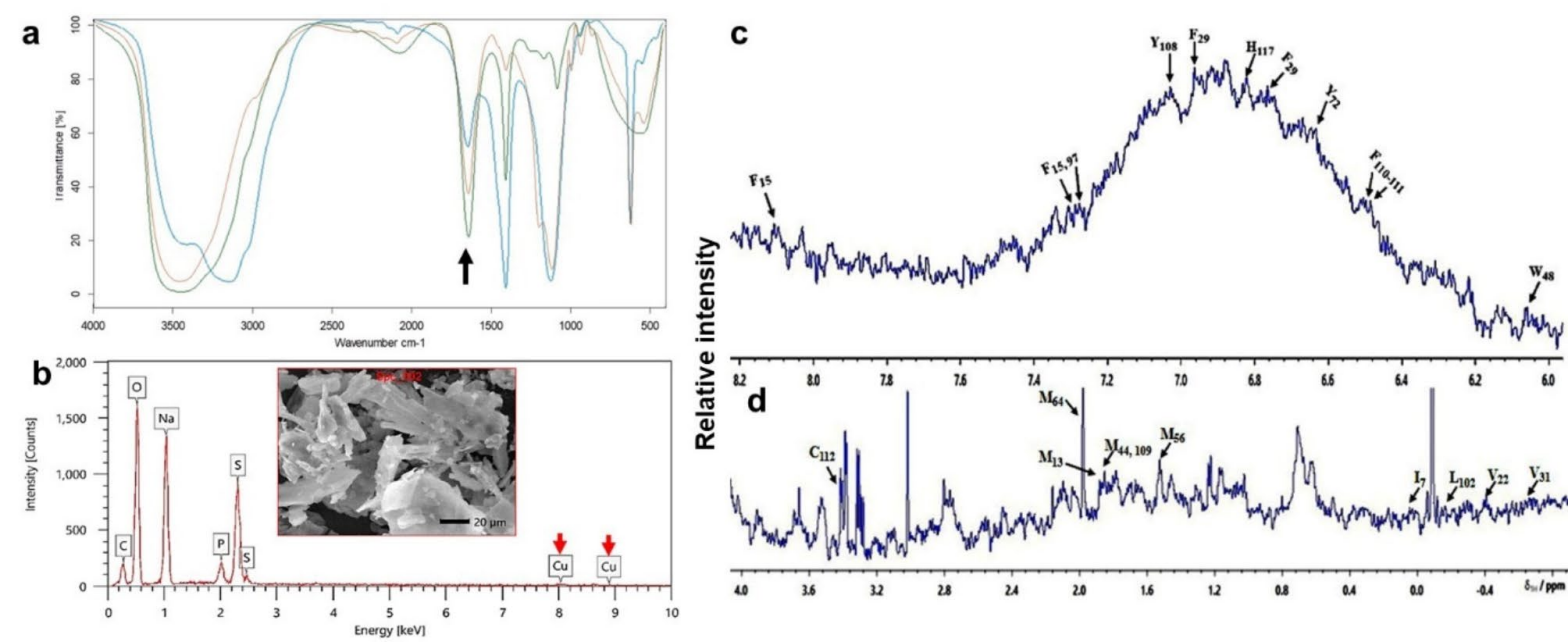
Characterization of purified azurin from *Pseudomonas aeruginosa* strain 105. **(a)** FTIR spectrum. **(b)** EDX analysis. **(c) (d)** ¹H NMR spectrum obtained at 500 MHz.

FTIR spectroscopy was employed to characterize the functional groups present in the partially purified azurin protein **(**Fig. 5a**)**. The prominent peaks observed at 1657–1650 cm⁻¹ and 1550–1540 cm⁻¹ corresponded to the amide I and II bands, characteristic feature of proteins arising from carbonyl group vibrations. Additionally, the broadband observed between 1400 and 1000 cm^− 1^ can be attributed to C-N stretching and C-H bending vibrations in the protein backbone. The strong N-H stretching bands observed at 3439 cm⁻¹ and 3429 cm⁻¹ indicate hydrogen bonding.

### Elemental analysis by EDX spectroscopy

EDX was employed to determine the elemental composition of the purified azurin **(**Fig. 5b**)**. The EDX spectrum revealed a copper-rich protein, as evidenced by two strong peaks at approximately 8.0 keV and 9.0 keV. The mass ratio of these peaks (0.70) is consistent with the characteristic copper signature in the active site of azurin. Additionally, the presence of sulfur (mass ratio 11.15) likely originates from cysteine (Cys) and methionine (Met) residues.

### ^1^H NMR analysis of purified azurin

The ^1^H NMR spectrum of the purified azurin fraction (Fig. 5c-d) revealed signals corresponding to various functional groups. Aliphatic protons resonated in the range of δ 0.0-3.4 ppm. Notably, specific peaks at δ 0.075 ppm and δ 3.40 ppm were assigned to the side chains of methionine (Met121) and cysteine (Cys112), respectively. A single peak at δ 6.78 ppm indicated the presence of an aromatic amino acid resonance, likely due to phenylalanine (Phe97). Additionally, multiple peaks between δ 5.2 ppm and δ 2.1 ppm were attributed to various aliphatic hydrogens in the protein structure.

### Antimicrobial activity and MIC of purified azurin

**Fig. 6:**
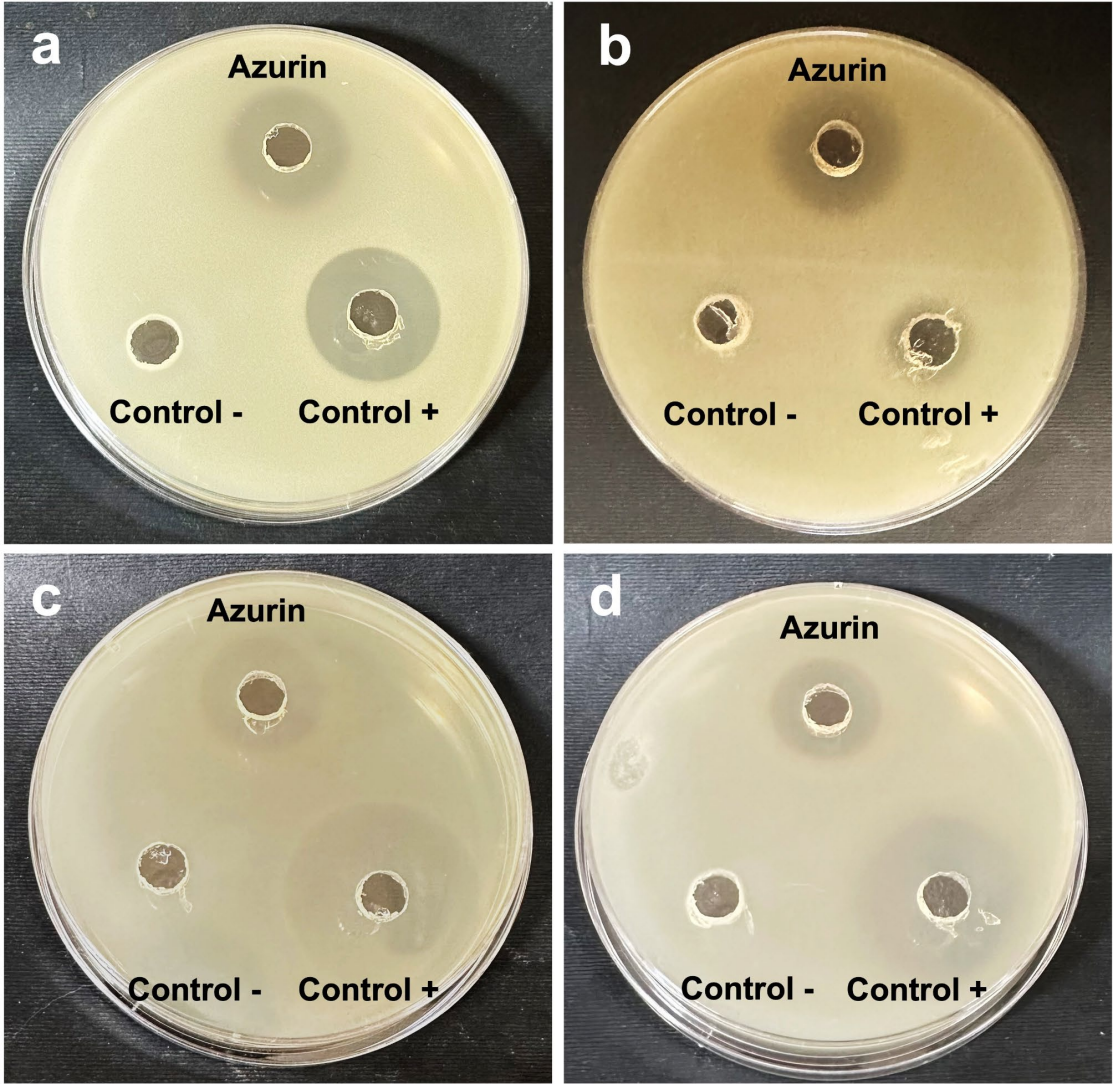
Antimicrobial activity of purified azurin against various bacterial strains. **(a)***Staphylococcus aureus* (ATCC 25923), **(b)***Bacillus subtilis* (ATCC 6633), **(c)***Escherichia coli* (ATCC 8739), and **(d)***Klebsiella pneumoniae* (ATCC 13883). Ampicillin (10 mg/mL; Fisher Bioreagents) was used as a positive control, and sterile saline solution (0.9% w/v) served as a negative control. Inhibition zones were measured in mm after 24 h of incubation.

The antimicrobial potential of purified azurin was assessed qualitatively against Gram-negative bacteria (*E. coli* and *K. pneumoniae*) and Gram-positive bacteria (*S. aureus* and *B. subtilis*) using well diffusion assay (Fig. 6). Azurin exhibited growth inhibition against all tested bacterial strains. The diameter of the inhibition zones ranged from 18.0 mm to 23.0 mm. *B. subtilis* displayed the highest susceptibility to azurin with an inhibition zone of 23.0 mm, while *K. pneumoniae* was the least affected strain with an inhibition zone of 18.0 mm. Further investigation was performed using the Minimum inhibitory concentration (MIC) assay. The MIC values of purified azurin were 64, 128, 128, and 512 µg/mL for *E. coli*, *K. pneumoniae*,* S. aureus*, and *B. subtilis*, respectively.

### Cytotoxicity and anticancer activity

**Fig. 7:**
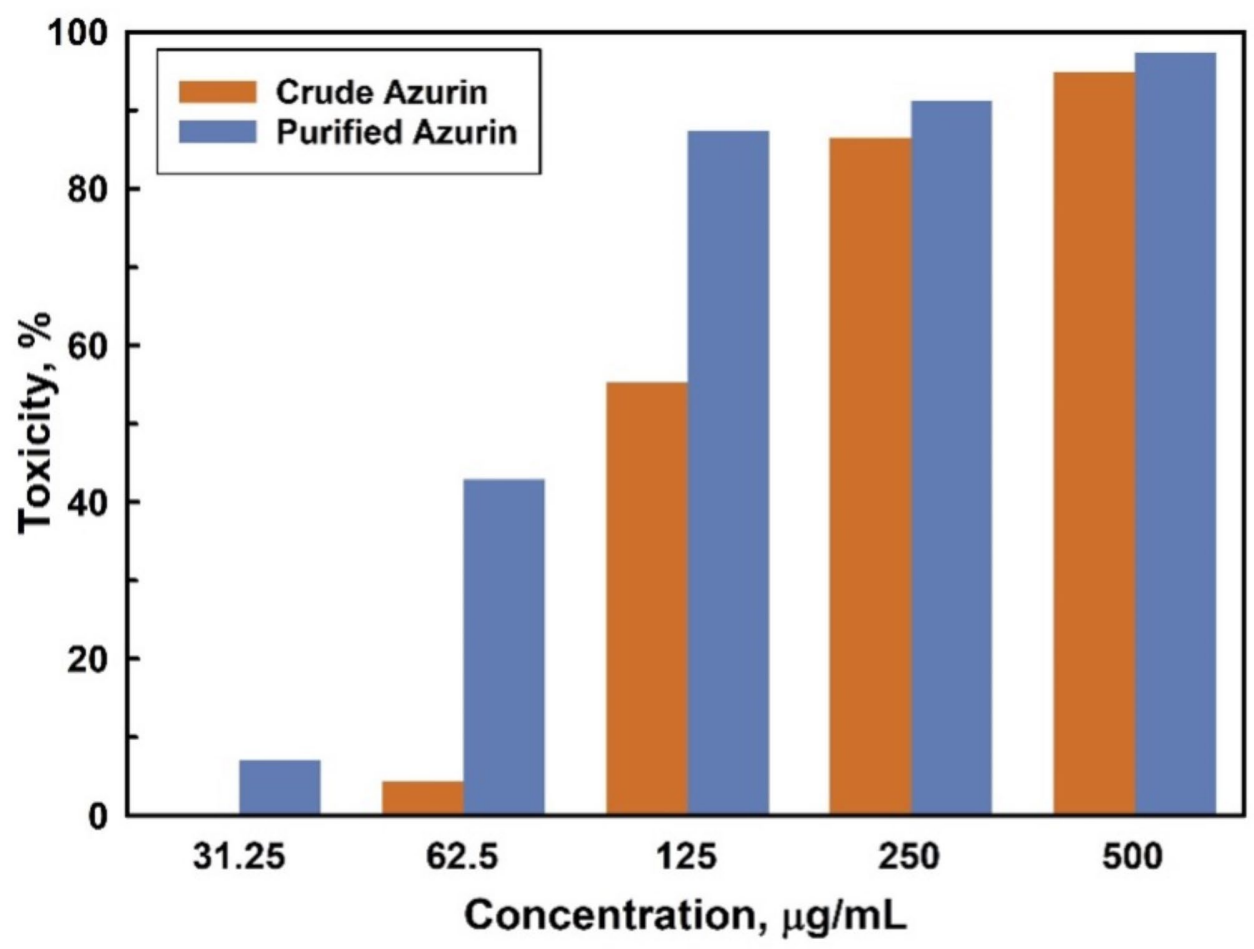
Dose-dependent anticancer activity of crude and purified azurin against MCF7 breast cancer cells as measured by MTT assay.

The cytotoxicity of ammonium sulfate-precipitated (crude) azurin was evaluated in vitro against the normal epithelial kidney cell line, VERO cells, using an MTT assay. Azurin exhibited no cytotoxic effect on the VERO cells, with an IC_50_ value of 400.13 ± 0.70 µg/mL. Cell viability remained high at concentrations below 250 µg/mL, indicating azurin’s safety towards kidney cells. Conversely, both ammonium sulfate-precipitated and purified azurin displayed dose-dependent anticancer activity against the MCF7 breast cancer cell line (Fig. 7). Increasing azurin concentration resulted in a significant decline in cell proliferation and morphological changes in the cancer cells (Supplementary Fig. S3). The IC_50_ values were determined to be 117.99 ± 1.12 µg/mL and 77.81 ± 0.56 µg/mL for crude and purified azurin, respectively. Notably, purified azurin induced significant cell death, starting at around 62.50 µg/mL.

### Cell cycle and apoptosis analysis of purified azurin

**Fig. 8:**
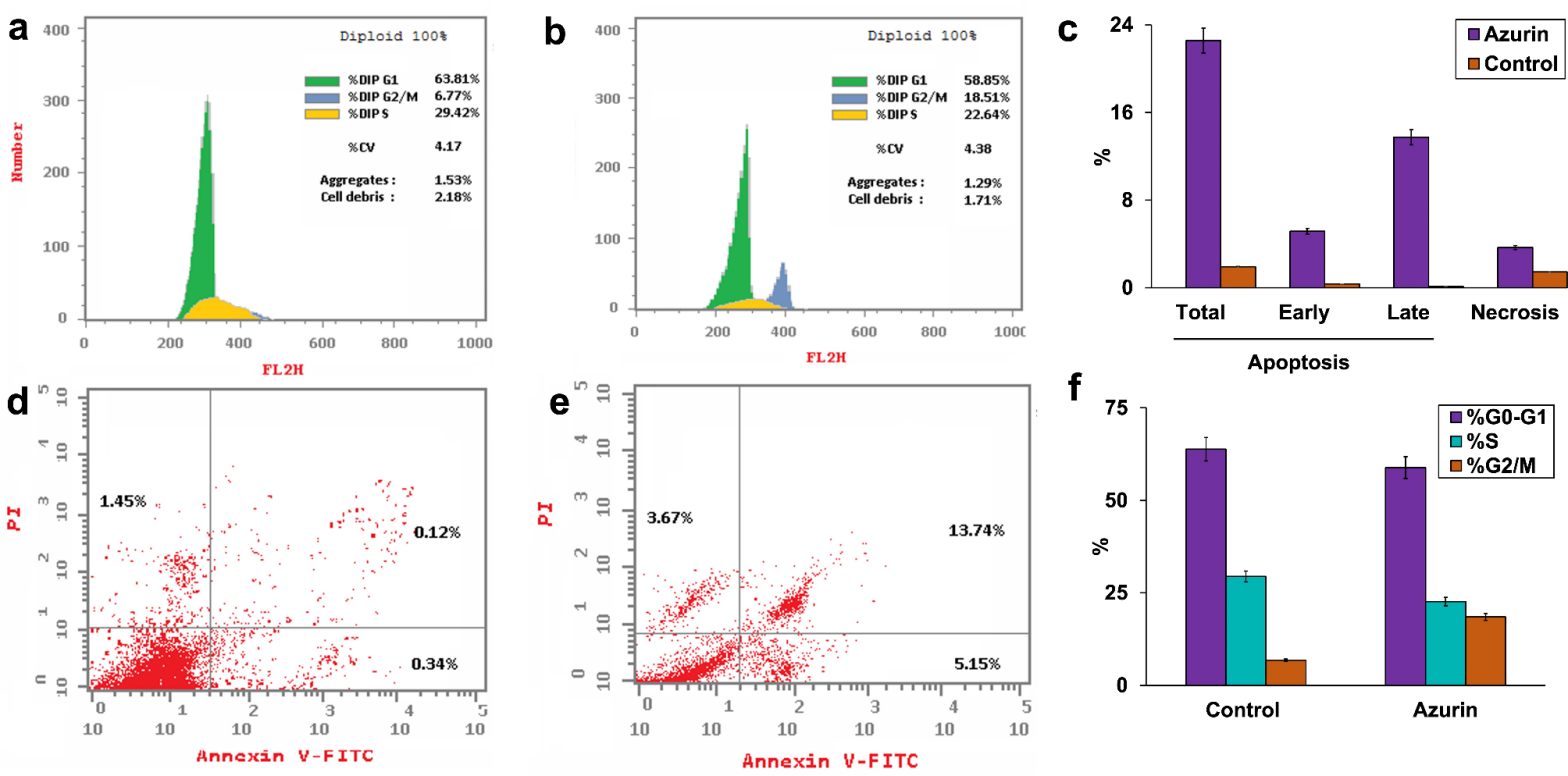
Effect of purified azurin on MCF7 breast cancer cells. **(a-c)** Apoptosis analysis by flow cytometry with Annexin V-PI staining. **(a)** Untreated control cells. **(b)** Cells treated with the IC₅₀ concentration of purified azurin. **(c)** Quantification of apoptotic cell population (early + late apoptosis). **(d-f)** Cell cycle analysis by propidium iodide staining. **(d)** Untreated control cells. **(e)** Cells treated with the IC₅₀ concentration of purified azurin. **(f)** Cell cycle distribution of control and azurin-treated cells.

The effect of purified azurin on the apoptotic response of MCF7 breast cancer cells was investigated using flow cytometry and Annexin V-PI staining to identify different apoptotic stages. Flow cytometry analysis revealed a significant increase in the apoptotic population compared to untreated control cells (Fig. 8a-c). Treatment with purified azurin resulted in 5.15%, 13.74%, and 3.67% of MCF7 cells undergoing early apoptosis, late apoptosis, and necrosis, respectively. Notably, total apoptosis in azurin-treated cells increased approximately 12-fold compared to the control. Additionally, flow cytometry analysis with propidium iodide staining (Fig. 8d-f) indicated that azurin treatment induced cell cycle arrest at the G2/M phase.

## Discussion

The high morbidity and mortality associated with cancer highlight the critical need for novel therapeutic options^28^. Bacterial cuperdoxins, a class of metalloproteins, represent a promising avenue for cancer therapy. Azurin, a specific member of this family, has demonstrated significant potential due to its preferential targeting of cancer cells and the lack of observed adverse effects in vivo studies^20,29,30^. Building on these findings, the present study investigated the production of azurin from a local isolate of *P. aeruginosa* strain 105. The isolated azurin was then subjected to physicochemical characterization and in vitro evaluation for its anticancer and antimicrobial activities.

Azurin biosynthesis is typically associated with a single, conserved azurin-encoding gene in beta-proteobacteria, suggesting its critical functions^16,31^. Prior studies have identified the azurin gene in environmentally sourced *P. aeruginosa* strains^32,33^. Consistent with these reports, our isolate 105 exhibited azurin expression at the RNA level, potentially due to the presence of a functional azurin promoter within its genome^31^.

Analysis of *P. aeruginosa* 105 growth kinetics revealed that azurin production was growth-associated, reaching its maximum during the stationary phase. The presence of copper sulfate and potassium nitrate in the culture medium further enhanced azurin production. Previous studies have demonstrated that supplementation of copper sulfate and potassium nitrate promotes azurin synthesis in *P. aeruginosa* MTCC 2453, likely due to their roles in maintaining azurin stability^34,35^.

The functional groups of azurin were analyzed using FTIR spectroscopy, providing a valuable model for studying proteins with characteristic β-barrel structures. As shown in Fig. 5a, the FTIR spectrum displays characteristic bands representing α-helix, β-sheet, β-turn, and random coil conformations within the amide I (1700–1600 cm⁻¹) and amide II (1560–1500 cm⁻¹) regions^35,36^. The amide I band, primarily arising from C = O stretching vibrations in peptide linkages, is especially sensitive to protein secondary structure. It is well established that α-helical conformations exhibit amide I and II bands between 1657 and 1650 cm⁻¹ and 1550–1540 cm⁻¹, respectively^32,37–39^. For β-sheets, these bands typically occur between 1635 and 1615 cm⁻¹ for amide I and 1535–1520 cm⁻¹ for amide II^40^. An additional peak at 1402.49 cm⁻¹ falls slightly outside the typical amide II range but may indicate complementary bending vibrations associated with the β-sheet structure, adding evidence to the β-barrel conformation of azurin. The strong N-H stretching bands observed at 3439 cm⁻¹ and 3429 cm⁻¹ indicate hydrogen bonding, further supporting the protein’s folded, stable conformation. These findings support that azurin folds into a stable, three-dimensional β-barrel structure organized in a double-wound Greek key topology^41^. The distinct β-sheet amide I band at 1635.94 cm⁻¹ and the hydrogen-bonded N-H stretching bands further confirm its characteristic secondary structure and folded conformation.

EDX spectroscopy confirmed the presence of a type I copper ion within the azurin structure, consistent with previous reports describing *P. aeruginosa* azurin as a small (128-residue), blue-copper protein harboring a β-barrel structure that coordinates a redox-active copper [Cu(II)/Cu(I) pair]^15^. Furthermore, the EDX analysis detected sulfur, aligning with the identification of two sulfur-containing ligands (Cys112 and Met121) coordinating the copper and facilitating disulfide bond formation, as reported by Schulz et al.^42^.

NMR spectroscopy is one of the principal techniques used for the identification, quantification, and monitoring of biological and biomedical samples^43^. The spectra of biomolecules as proteins are relatively complex, due to the presence of a large number of protons in protein molecules^44,45^, although azurin is a single-domain protein^46^. The various peaks in the spectrum correspond to different protons in the azurin molecule that could be affected by various variables^47^. The positions of these peaks depend on the chemical environment of each proton and are early assigned for azurin^47–49^. The assignments of some of the peaks are indicated in the spectrum. The protons of the aromatic residues showed peaks at around 6.05 ppm assigned to the C2 proton of the tryptophane (W48), while the peak at around 7.7 ppm is the C4 proton of the tyrosine residue (Y72), similarly, the proton of the phenylalanine residues (F15-F111) of different chemical environments (C2/C6, C3/C5, C4, and C7) are distributed from approximately 6.5 ppm to 8.09 ppm (Fig. 5c). The C2/C4 protons of the histidine residue (H117) are assigned at about 6.9 ppm. On the other hand, aliphatic protons for valine (V31, V22) are assigned around − 0.60 and − 0.4 ppm. Peaks at around 1.6 to 2.05 ppm are assigned to the ε-protons of the methyl groups of the five methionine residues of azurin (M13, 44, 64, 56, and M109). The peak for α-protons of the cysteine residue (C112) is assigned at around 3.4 ppm **(**Fig. 5d**)**; this residue is very close to the copper-binding site^49^.

Interestingly, azurin extracted from *P. aeruginosa* 105 showed potential antimicrobial activity against *E. coli*, *K. pneumoniae*, *S. aureus*, and *B. subtilis*. Azurin exerts antimicrobial activity against bacteria and this activity is attributed to several mechanisms. It is structurally similar to cationic antimicrobial peptides, which may bind directly to negatively charged bacterial cell membranes, causing disruptions in their integrity and pore formation^50^. The loss of membrane integrity causes cell lysis and death^51^. Moreover, a previous study reported that recombinant azurin revealed a high inhibitory effect against bacterial growth and biofilm formation of *Salmonella typhi* and *Vibrio cholera*^52^. Further study revealed the efficacy of azurin as a peptide-based-anti-adhesion agent that probably interacts with bacterial receptors and ultimately reduces bacterial adhesion and pathogenesis^53^.

Building on prior research demonstrating the cytotoxicity of copper-containing redox proteins from *P. aeruginosa* against human breast cancer cells^54^, we evaluated the anticancer potential of our purified azurin. Notably, azurin treatment has been shown to induce apoptosis in over 50% of MCF7 breast cancer cells, with no observed adverse effects in vivo studies^20,30,55^.

Cytotoxicity assay revealed a selective effect of azurin. While non-toxic to VERO cells (IC_50_ > 400 µg/mL), azurin from *P. aeruginosa* 2453 significantly inhibited the growth of both T-47D and ZR-75-1 breast cancer cell lines (IC_50_ = 72.0 ± 3.0 µg/mL)^56^. This observed activity is comparable to that obtained for azurin produced in this study (IC_50_ = 77.81 ± 0.56 µg/mL). Furthermore, our results demonstrate improved efficacy compared to previous studies using the same cell lines (IC_50_ = 105 µg/mL)^57^ and purified azurin from *P. aeruginosa* SSj (IC_50_ = 102 µg/mL)^32^.

Azurin enhances the effectiveness of various anticancer drugs by increasing cancer cell sensitivity to treatment. Azurin improves treatment response in lung, breast, cervical, colon, and oral squamous carcinoma cells, making them more susceptible to drugs like gefitinib, erlotinib, paclitaxel, doxorubicin, 5-fluorouracil, and etoposide. This effect suggests azurin could be valuable in overcoming drug resistance in certain cancer types^6,30,58,59^.

Furthermore, azurin from *P. aeruginosa* MTCC 2453 demonstrated promising in vitro and in vivo antitumor activity against Dalton’s lymphoma, highlighting its potential therapeutic application^56^. Similarly, studies using the p28 peptide derived from azurin (Leu50-Asp77) reported significant reductions in ZR-75-1 breast cancer cells^60^.

The anticancer potential of azurin is attributed to the p28 domain (Leu50-Asp77), a peptide responsible for cellular uptake. This domain disrupts breast cancer cell proliferation and induces apoptosis via the mitochondrial pathway^54,61^. Our study confirms a significant increase in total cell apoptosis of MCF7 cells treated with azurin compared to the control. Additionally, a decrease in DNA content (% G0-G1) was observed, corroborating previous reports on the cell cycle arrest induced by azurin treatment^35,57^ identifying another C-terminal domain in azurin that facilitates binding to EphB2 receptors, potentially contributing to tumor growth inhibition.

## Conclusion

This study focused on locally isolated *Pseudomonas aeruginosa* strains from Egypt, specifically strain 105, which demonstrated significant azurin synthesis. This study not only confirms azurin’s anticancer potential but also explores its antimicrobial properties and dives into the structural characterization of the blue copper protein. The partially purified azurin exhibited promising anticancer activity against breast cancer cell lines and antimicrobial properties against various bacterial strains. Prospects are the production of azurin at a larger scale through economically feasible approaches to possibly establish a local biotech platform for its production. Furthermore, in vivo studies are warranted to evaluate the therapeutic efficacy of azurin in cancer treatment. This research holds significant potential to contribute to the development of novel, efficacious anticancer drugs with reduced side effects either alone or in combination with established therapies.

## Methods

### Sample collection and *P. aeruginosa* isolation

In March 2021, samples were collected from different locations, including soil, fresh, and marine water (Egypt). For the isolation of *P. aeruginosa*, Cetrimide agar plates (HiMedia, India) were used. After aerobic incubation at 32 °C for 24 h, single bacterial colonies were selected and purified through two consecutive rounds of streaking on fresh Cetrimide agar plates. Confirmed *P. aeruginosa* isolates were then stored at -20 °C in LB broth supplemented with 15% (v/v) glycerol for further analysis.

### Genomic DNA extraction

Genomic DNA was extracted from a 1.5 mL aliquot of an overnight bacterial culture (grown for 18 h at 37 °C in LB broth) using the GeneJET Genomic DNA Purification Kit (Thermo Fisher Scientific, UK) according to the manufacturer’s protocol. The concentration and purity of the isolated DNA were assessed using a NanoDrop ND-2000 spectrophotometer (Thermo Fisher Scientific, USA) and 1% (w/v) agarose gel electrophoresis.

### Biochemical and molecular identification

A single isolate was selected for identification. Biochemical testing was performed using a VITEK 2 GN ID card (BioMérieux, France). Additionally, molecular identification was performed through 16S rRNA gene sequencing. Purified genomic DNA was used as a template for PCR amplification of the 16S rRNA gene using the specific primers 27 F (5’-AGAGTTTGATCCTGGCTCAG-3’) and 1492R (5’-CGGTTACCTTGTTACGACTT-3’). The amplified 16S rRNA amplicon from the selected isolate was sequenced using an ABI 3730XL DNA Analyzer (ACGT, Germany). The obtained sequence was then analyzed for similarity using the National Center for Biotechnology Information (NCBI) BLAST database (https://blast.ncbi.nlm.nih.gov/). Phylogenetic tree construction was performed using the Maximum Likelihood method in bootstrapping (1000 replicates) implemented in the MEGA software (Version 11.0)^62,63^.

### Production and extraction of azurin

#### Cultivation of *P. aeruginosa* strain 105

The culture medium used in this study was formulated as follows (g/L): peptone 10.0; yeast extract 7.0; NaCl 2.5; KH_2_PO_4_ 6.4; Na_2_HPO_4_ 3.6; KNO_3_ 0.5; and CuSO_4_.5H_2_O 0.005^35^. *P. aeruginosa* strain 105 was cultured in 250 mL flasks containing 100 mL of the production medium. The flasks were inoculated with 1% (v/v) of an overnight culture (grown for 18 h). Flasks were incubated at 37 °C and 150 rpm in an orbital shaking incubator. Cell growth was monitored by obtaining samples at various time points (12–48 h). After incubation, cells were harvested by centrifugation at 4000 rpm at 4 °C, and cell fresh weight was determined.

#### Azurin extraction

Harvested cells (2.0 g) were resuspended in 10 mL of 0.02 M potassium phosphate buffer (pH 7.0; Merck, Germany). Cell disruption was achieved by ultrasonication (Sonopuls, Germany) for 1.0 min/mL, followed by centrifugation to remove cell debris. The total protein concentration in the supernatant was quantified using the Bradford method by measuring absorbance at 595 nm^64^. The presence of azurin was confirmed by measuring the absorbance ratio (Abs_625_/Abs_280_) using a UV-Vis spectrophotometer (Jenway 6305, UK) and ensuring it falls within the reported range for azurin (0.53–0.58)^65^.

### Detection of azurin

#### Detection of azurin by CuCl2 method

The presence of azurin in the cell extract was confirmed using the CuCl₂ method^66^. Briefly, 8 µL of a 0.2 M CuCl₂ solution was added to 3 mL of the cell lysate. The mixture was incubated at room temperature for 24 h. A gradual color change to deep blue was indicative of azurin, a blue copper protein.

#### Detection of azurin gene in chromosomal DNA

The nucleotide sequences of azurin (*azu*) genes from representative 11 *P. aeruginosa* strains were retrieved from the NCBI database and aligned using CLC Workbench software (Version 7.7.3). Based on the multiple sequence alignment of consensus regions identified in azurin genes, primers flanking the *azu* gene were designed manually: *azuF* (5’-ATGCTACGTAAACTCGCTGCGGTATCC-3’) and *azuR* (5’-TCAGGGTCAGGGTGCCCTTCATCAG-3’). The primers were synthesized by Willowfort (UK) and resuspended in nuclease-free water to a final concentration of 10 pmol/µL. PCR amplification of the *azu* gene was performed using 2X TOPsimple PreMIX-nTaq DNA polymerase (Enzynomics, Korea, #P610T) according to the manufacturer’s instructions. Each 20 µL PCR reaction mixture contained: 10 µL of Master-mix, 1 µM each of the forward and reverse primers (*azuF* and *azuR*), 1 µL of template DNA (50–100 ng/µL), and nuclease-free water to reach the final volume. The thermal cycling program consisted of an initial denaturation step at 94 °C for 4 min, followed by 35 cycles of denaturation (94 °C for 30 s), annealing (60 °C for 30 s), and extension (72 °C for 1 min). A final extension step at 72 °C for 5 min was included. The PCR reactions were carried out in a TECHNE TC-3000 thermal cycler.

The amplicons were resolved by electrophoresis on a 1% (w/v) agarose gel prepared in 1X Tris-Borate-EDTA (TBE) buffer. The gel was stained with a 5% (w/v) ethidium bromide solution and visualized using a UV transilluminator. The amplicon was sequenced bidirectionally, and the physical parameters of the resulting sequence were determined with the ProtParam-ExPASY Proteomics Tool (https://web.expasy.org/protparam/). The azurin-translated sequence was aligned using the NCBI database with the BLASTp tool, and the evolutionary analysis of the azurin-translated protein sequence was inferred using the Maximum Likelihood method and JTT matrix-based model^67^. Evolutionary analysis was conducted using MEGA software (Version 11.0)^63^.

### RNA extraction and cDNA synthesis

Total RNA was isolated from a 1.5 mL aliquot of an overnight bacterial culture (grown for 18 h in an azurin production medium) using the ABT Total RNA Extraction Kit (Applied Biotechnology, Egypt) according to the manufacturer’s protocol. The purity and quality of the extracted RNA were assessed by measuring the absorbance ratio (Abs_260_/Abs_280_). cDNA synthesis was then performed using the ABT H-Minus cDNA Synthesis Kit (Applied Biotechnology, Egypt). The reaction mixture contained approximately 100 ng of total RNA template, 2 µL of oligo (dT)18 primer, 4 µL of 5X reaction buffer, 0.5 µL of reverse transcriptase enzyme, 2 µL of 10 mM dNTP mix, and nuclease-free water to reach a final volume of 13.5 µL. The reaction was incubated at 25 °C for 5 min, followed by 42 °C for 1 h, and finally terminated at 70 °C for 5 min. The synthesized cDNA was then used for conventional PCR amplification.

### Extraction of crude azurin and purification

The cell lysate obtained from sonicated *P. aeruginosa* cells was subjected to ammonium sulfate precipitation. To achieve a final concentration of 70% (w/v), ammonium sulfate was slowly added to the lysate with continuous stirring at 4 °C. The precipitated proteins were collected by centrifugation, and the supernatant was discarded. The protein pellet was resuspended in a suitable buffer (e.g., phosphate-buffered saline, PBS) and dialyzed overnight at 4 °C using a Float-A-Lyzer G2 Dialysis Device (Spectrum Laboratories) with a 0.5-1.0 kDa MWCO membrane to remove residual salts. The dialyzed protein solution was then concentrated using a Vivaspin 20 centrifugal concentrator (Sartorius, Germany) with a 30 kDa MWCO membrane. The concentrated crude protein solution was loaded onto a column packed with carboxymethyl Sephadex (CM-Sephadex; WINLAB, UK), a cation exchange chromatography medium. The column was equilibrated with phosphate citrate buffer (pH 6.0). Unbound proteins were removed by washing the column with 2-bed volumes of the equilibration buffer. Bound azurin was eluted using a stepwise sodium chloride gradient (0, 0.2, 0.3, and 0.5 M) in the same buffer at a flow rate of 1.0 mL/min. Fractions were collected and monitored by measuring their absorbance at 280 nm using a UV-Vis spectrophotometer (Jenway 6305, UK). Fractions containing azurin, as identified by their absorbance peak, were pooled, lyophilized in a freeze dryer (Christ, Germany), and stored at -20 °C for further analysis.

### Structural characterization of azurin

#### Polyacrylamide gel electrophoresis (SDS-PAGE)

The purity and molecular weight of the eluted protein fractions were determined by sodium dodecyl sulfate-polyacrylamide gel electrophoresis (SDS-PAGE) using a 15% Tris-glycine gel^68^. A pre-stained 10–170 kDa protein marker (Maestrogen, China) was used as a reference for molecular weight estimation.

#### Fourier-transform infrared spectroscopy (FTIR)

Approximately 10 mg of the lyophilized, purified azurin fraction was finely ground with potassium bromide (KBr) crystals. The mixture was then pressed into a disc for Fourier-Transform Infrared (FTIR) spectroscopy analysis using a Bruker Tensor 27 spectrometer (Germany). The FTIR spectrum was recorded at room temperature in the mid-infrared region (400–4000 cm⁻¹). This analysis aimed to identify the functional groups present in the purified azurin molecule.

#### Energy-dispersive X-ray spectroscopy (EDX) analysis

The elemental composition of the purified azurin fraction was determined by energy-dispersive X-ray spectroscopy (EDX) at an accelerating voltage of 20.0 kV using a scanning electron microscope (SEM; JSM-IT 200, JEOL, Japan) located at the Electron Microscope Unit, Alexandria University, Egypt.

#### ^1^H nuclear magnetic resonance (NMR) spectroscopy

For structural characterization of the purified azurin, proton nuclear magnetic resonance (^1^H NMR) spectroscopy was performed at a spectrometer frequency of 500 MHz (JNM-ECZ500R, JEOL, Japan) located at the Central Lab, Faculty of Science, Alexandria University (Alexandria, Egypt). Approximately, 20 mg of the lyophilized, purified azurin fraction was completely dissolved in deuterated water (D2O) for NMR analysis. The acquired ¹H NMR spectrum was used to determine the chemical shift (δ) values reported in parts per million (ppm).

### Biological properties of purified azurin

#### Antibacterial activity

##### Well diffusion assay

The antibacterial activity of the purified azurin was evaluated against Gram-negative bacteria *Escherichia coli* (ATCC 8739), *Klebsiella pneumoniae* (ATCC 13883), and Gram-positive bacteria *Staphylococcus aureus* (ATCC 25923), and *Bacillus subtilis* (ATCC 6633) using the agar well diffusion method. The standard bacterial pathogens were obtained from the Clinical Pathology Department, Faculty of Medicine, Alexandria University, Egypt. Bacterial cultures were adjusted to a turbidity of approximately 0.5 McFarland standard (corresponding to ~ 10^6^ CFU/mL) and swabbed onto Mueller-Hinton agar (MHA; Oxoid, UK) plates. Wells with a diameter of 6.0 mm were punched into the agar and filled with 100 µL of sterile azurin solution at a defined concentration (10 mg/mL). The plates were then incubated at 37 °C for 24 h. Ampicillin (10 mg/mL; Fisher Bioreagents) served as a positive control, while sterile saline solution (0.9% w/v) was used as a negative control. Antibacterial activity was determined by measuring the diameter of the inhibition zone around each well. The experiment was performed in duplicate for each bacterial strain.

##### Minimum inhibitory concentration (MIC)

Minimum inhibitory concentration (MIC) was determined using the broth micro-dilution method according to the Clinical and Laboratory Standards Institute^69^. In 96-well polystyrene microtiter plates, MHB was used to prepare sequential 2-fold serial dilutions ranging from 1,024 to 1 µg/mL. The plates were subsequently inoculated with 50 µL of a bacterial suspension adjusted to 10^6^ CFU/mL, and then the plates were incubated at 37 °C aerobically for 24 h. MIC was defined as the lowest concentration, showing no visible bacterial growth. All experiments were performed in triplicate.

##### Cytotoxicity and anticancer activity assay

The cytotoxicity of the purified azurin was evaluated against normal human epithelial kidney cells (VERO cells; ATCC CCL-81) and the MCF7 human breast cancer cell line (ATCC HTB-22) using the MTT assay^70^. Briefly, cells were seeded at a density of 10^5^ cells per well in a 96-well plate and incubated at 37 °C for 24 h. Serially diluted azurin solutions were prepared in RPMI 1640 medium (Sigma-Aldrich, USA) supplemented with 2% fetal bovine serum (FBS). Aliquots of 100 µL from each dilution were added to the respective wells containing pre-seeded cells. After incubation with the azurin dilutions, 20 µL of MTT solution (5.0 mg/mL in PBS) was added to each well. The plates were then gently mixed and incubated for an additional 4 h at 37 °C in a humidified incubator with 5% CO_2_ to allow for MTT formazan formation. Following incubation, the medium was carefully removed, and the formed formazan crystals were dissolved in 100 µL of dimethyl sulfoxide (DMSO) per well. The absorbance of the dissolved formazan was measured at 560 nm using a microplate reader.

##### Cell cycle analysis by propidium iodide staining

The effect of purified azurin on the cell cycle distribution of MCF7 cells was determined using propidium iodide (PI) staining and flow cytometry analysis. MCF7 cells were treated with a defined concentration of purified azurin (77.81 ± 0.56 µg/mL) for 48 h. Following treatment, cells were harvested, fixed with 66% ethanol (v/v) for at least 2 h at 4 °C, and stored until analysis. Fixed cells were centrifuged, washed with PBS, and resuspended in a staining solution containing propidium iodide (PI) and RNase A (ab139418, Abcam). The cell suspension was incubated in the dark at 37 °C for 30 min to allow for PI staining of cellular DNA. Finally, stained cells were analyzed using a flow cytometer (BD FACSCalibur, USA) to quantify the cell population distribution across the cell cycle phases (G0/G1, S, and G2/M).

## Electronic supplementary material

Below is the link to the electronic supplementary material.

Supplementary Material 1

Supplementary Material 2

## Data Availability

Sequence data that support the findings of this study has been deposited in the GenBank with the primary accession code PP431212 and PP848219.
