# Supplementary material for: Azurin a potent anticancer and antimicrobial agent isolated from a novel Pseudomonas aeruginosa strain
Source: Sci Rep. 2025 Jan 30;15:3735. doi: 10.1038/s41598-025-86649-w (PMC11782508; doi:10.1038/s41598-025-86649-w)
Supplement: Supplementary file 1 — Supplementary Material 1 [file 41598_2025_86649_MOESM1_ESM.pdf]

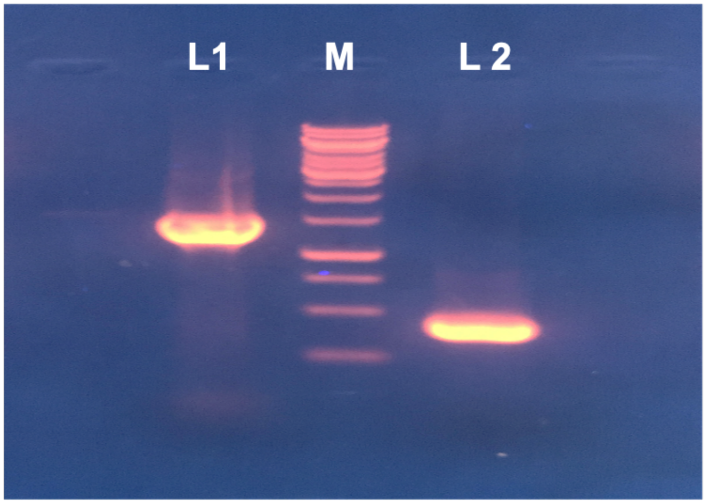

Supplementary Figure S1. **Agarose gel electrophoresis for detection of the azurin gene. Lane M: 1 kb Gene Ruler (Fermentas, UK), Lane 1: PCR amplicon of 16S rRNA gene of isolate105, Lane 2: PCR amplicon of the azurin gene (*azu*) from isolate 105 with the expected size of 442 bp.**

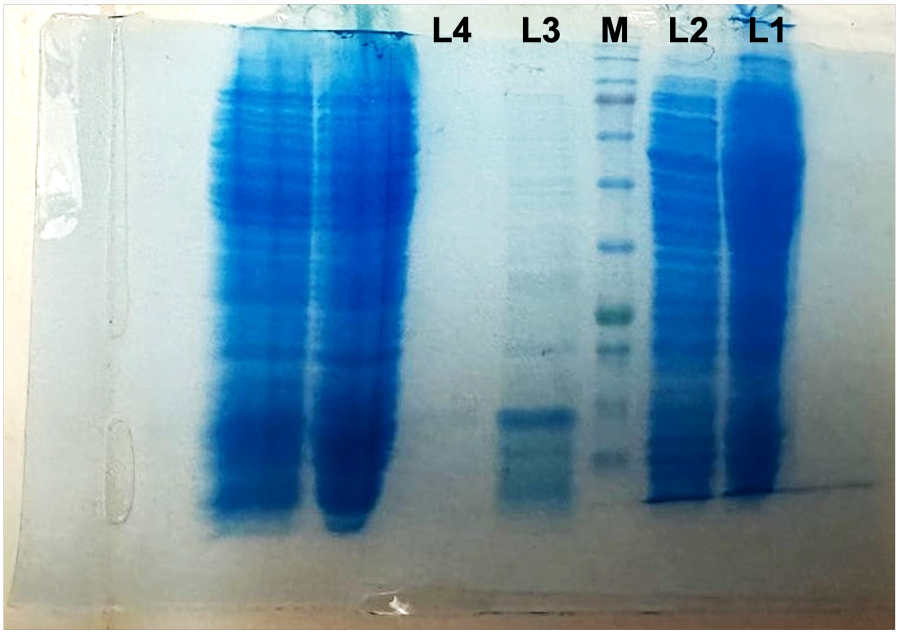

Supplementary Figure S2. SDS-PAGE of azurin fractions. Lane M: protein marker (Maestrogen, China), Lane 1: ammonium precipitated protein, Lane 2: concentrated protein with a 30 kDa MWCO membrane, Lane 3: fraction 1 semi-purified azurin protein, Lane 4: purified azurin protein.

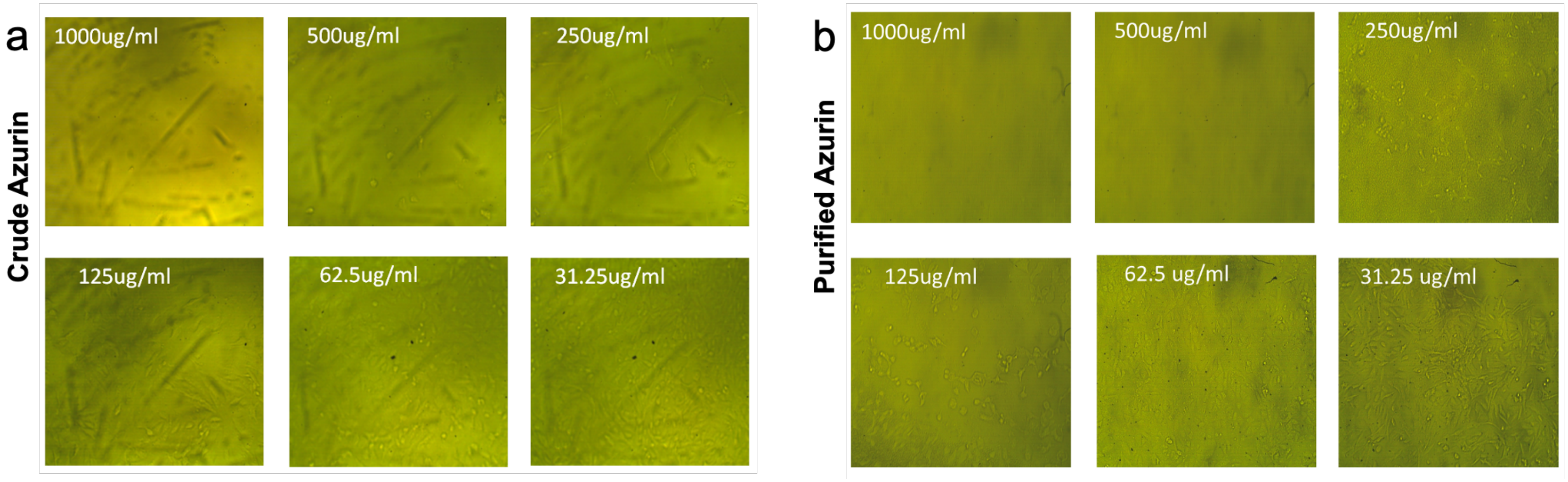

Supplementary Figure S3. Effect of azurin produced by *Pseudomonas aeruginosa* strain 105 on MCF7 breast cancer cell lines at different concentrations, (a) Crude azurin (b) Purified azurin.
