# Supplementary material for: Azurin a potent anticancer and antimicrobial agent isolated from a novel Pseudomonas aeruginosa strain
Source: Sci Rep. 2025 Jan 30;15:3735. doi: 10.1038/s41598-025-86649-w (PMC11782508; doi:10.1038/s41598-025-86649-w)
Supplement: Supplementary file 2 — Supplementary Material 2 [file 41598_2025_86649_MOESM2_ESM.docx]

**Supplementary Table 1. Phenotypic Characteristics of Potential *Pseudomonas aeruginosa* Isolates from Various Sources.**

| **Isolate Code** | **Source  of Isolation** | **Sample  Type** | **Gram stain Reaction** | **Haemolytic  Activity^8^** | **Growth  at 42 °C^9^** | **Pigment  Production^10^** |
| --- | --- | --- | --- | --- | --- | --- |
| **103** | El Nubaria**^1^** | Water | -ve | **α** | +++ | +++ |
| **104** | El Nubaria**^1^** | Water | -ve | **α** | +++ | +++ |
| **105** | El Nubaria**^1^** | Water | -ve | **α** | +++ | +++ |
| **201** | El Nubaria**^1^** | Soil | -ve | **α** | +++ | +++ |
| **202** | El Nubaria**^1^** | Soil | -ve | **α** | +++ | +++ |
| **203** | El Nubaria**^1^** | Soil | -ve | **α** | +++ | +++ |
| **301** | El Max**^2^** | Seawater | -ve | **α** | +++ | ++ |
| **303** | El Max**^2^** | Seawater | -ve | **α** | +++ | ++ |
| **304** | El Max**^2^** | Seawater | -ve | **α** | +++ | ++ |
| **501** | El Max**^2^** | Seawater | -ve | **α** | +++ | +++ |
| **502** | El Max**^2^** | Seawater | -ve | **α** | +++ | +++ |
| **D001** | Oily sewer**^3^** | Water | -ve | **α** | +++ | ++++ |
| **D004** | Oily sewer**^3^** | Water | -ve | **α** | +++ | ++ |
| **P3** | Pea soil**^4^** | Soil | -ve | **β** | +++ | - |
| **P4** | Pea soil**^4^** | Soil | -ve | **β** | ++ | - |
| **P5** | Bean’s soil**^5^** | Soil | -ve | **α** | +++ | - |
| **P6** | Bean’s soil**^5^** | Soil | -ve | **α** | +++ | ++++ |
| **P7** | El Marioutia**^6^** | Soil | -ve | **γ** | ++ | - |
| **P8** | El Marioutia**^6^** | Soil | -ve | **γ** | + | - |
| **P11** | Bahary**^7^** | Seawater | -ve | **α** | +++ | + |

^1^El Nubaria, Al Buhayrah governorate, Egypt. ^2^El Max, Alexandria governorate, Egypt. ^3^ Amreya, Alexandria governorate, Egypt. ^4,5^Botanical garden, Faculty of Science, Alexandria governorate, Egypt. ^6^El Mariotia, Giza governorate, Egypt. ^7^Bahary, Alexandria Governorate, Egypt. ^8^ Blood agar medium was used to test the haemolytic activity. ^9^ LB medium was used to test the growth at 42°C.^10^ Cetrimide agar plates medium was used to test the production of pigments at 42°C after 48 hours of incubation.

| **Supplementary Table 2. VITEK 2 biochemical characterization of six isolates identified as *Pseudomonas aeruginosa*.** | | | | | | |
| --- | --- | --- | --- | --- | --- | --- |
| **Biochemical reactions** | **Isolates’ codes** | | | | | |
|  | **D001** | **105** | **304** | **501** | **P6** | **P11** |
| Ala-Phe-Pro-ARYLAMIDASE(APPA) | **-** | **-** | **-** | **-** | **-** | **-** |
| ADONITOL(ADO) | **-** | **-** | **-** | **-** | **-** | **-** |
| L-Pyrrolydonyl-ARYLAMIDASE(PyrA) | **-** | **-** | **-** | **-** | **-** | **-** |
| L-ARABITOL (IARL) | **-** | **-** | **-** | **-** | **-** | **-** |
| D-CELLOBIOSE (dCEL) | **-** | **-** | **-** | **-** | **-** | **-** |
| BETA-GALACTOSIDASE(BGAL) | **-** | **-** | **-** | **-** | **-** | **-** |
| H₂S PRODUCTION(H₂S) | **-** | **-** | **-** | **-** | **-** | **-** |
| BETA-N-ACETYL-GLUCOSAMINIDASE(BNAG) | **-** | **-** | **-** | **-** | **-** | **-** |
| GLUTAMYL ARYLAMIDASE pNA (AGLTp) | **+** | **+** | **+** | **+** | **+** | **+** |
| D-GLUCOSE ( dGlu ) | **+** | **+** | **+** | **+** | **+** | **+** |
| GAMMA-GLUTAMYL-TRANSFERASE(GGT) | **+** | **+** | **+** | **+** | **+** | **+** |
| FERMENTATION/GLUCOSE(OFF) | **-** | **-** | **-** | **-** | **-** | **-** |
| BETA-GLUCOSIDASE (BGLU) | **-** | **-** | **-** | **+** | **-** | **-** |
| D-MALTOSE (dMAL) | **-** | **-** | **-** | **-** | **-** | **-** |
| D-MANNITOL (dMAN) | **+** | **+** | **-** | **-** | **+** | **+** |
| D-MANNOSE (dMNE) | **+** | **+** | **+** | **+** | **+** | **+** |
| BETA-XYLOSIDASE(BXYL) | **-** | **-** | **-** | **-** | **-** | **-** |
| BETA-Alanine arylamidase pNA (BAlap) | **+** | **+** | **+** | **+** | **+** | **+** |
| L-Proline ARYLAMIDASE (ProA) | **+** | **+** | **+** | **+** | **+** | **+** |
| LIPASE (LIP) | **-** | **+** | **+** | **+** | **+** | **+** |
| PALATINOSE (PLE) | **-** | **-** | **-** | **-** | **-** | **-** |
| Tyrosine ARYLAMIDASE (TyrA) | **+** | **+** | **+** | **+** | **+** | **-** |
| UREASE (URE) | **-** | **-** | **-** | **-** | **-** | **-** |
| D-SORBITOL (dSOR) | **-** | **-** | **-** | **-** | **-** | **-** |
| SACCHAROSE/SUCROSE (SAC) | **-** | **-** | **-** | **-** | **-** | **-** |
| D-TAGATOSE (dTAG) | **-** | **-** | **-** | **-** | **-** | **-** |
| D-TREHALOSE (dTRE) | **-** | **+** | **+** | **+** | **+** | **+** |
| CITARTE(SODIUM) (CIT) | **+** | **+** | **+** | **+** | **+** | **+** |
| MALONATE (MNT) | **+** | **+** | **+** | **+** | **+** | **+** |
| 5-KETO-D-GLUCONATE (5KG) | **-** | **-** | **-** | **-** | **-** | **-** |
| L-LACTATE alkalinization (ILATK) | **+** | **+** | **+** | **+** | **+** | **+** |
| ALPHA-GLUCOSIDASE (AGLU) | **-** | **-** | **-** | **-** | **-** | **-** |
| SUCCINATE alkalinization (SUCT) | **+** | **+** | **+** | **+** | **+** | **+** |
| Beta-N-ACETYL-GALACTOSAMINIDASE (NAGA) | **-** | **-** | **-** | **-** | **-** | **-** |
| ALPHA-GALACTOSIDASE (AGAL) | **-** | **-** | **-** | **-** | **-** | **-** |
| PHOSPHATASE (PHOS) | **-** | **-** | **-** | **-** | **-** | **-** |
| Glycine ARYLAMIDASE (GlyA) | **-** | **-** | **-** | **-** | **-** | **-** |
| ORNITHINE DECARBOXYLASE (ODC) | **-** | **-** | **-** | **-** | **-** | **-** |
| LYSINE DECAROXYLASE (LDC) | **-** | **-** | **-** | **-** | **-** | **-** |
| L-HISTIDINE assimilation (IHISa) | **-** | **-** | **+** | **-** | **-** | **-** |
| COURMARATE (CMT) | **+** | **+** | **+** | **+** | **+** | **+** |
| BETA-GLUCURONIDASE (BGUR) | **-** | **-** | **-** | **-** | **-** | **-** |
| O/129 RESISTANCE (comp.vbrio.) (O129R) | **+** | **+** | **+** | **+** | **+** | **+** |
| Glu-Gly-Arg-ARYLAMIDASE (GGAA) | **-** | **-** | **-** | **-** | **-** | **-** |
| L-MALATE assimilation (IMLTa) | **+** | **+** | **+** | **+** | **+** | **+** |
| ELLMAN(ELLM) | **+** | **-** | **-** | **-** | **-** | **-** |
| L-LACTATE assimilation (ILATa) | **+** | **+** | **+** | **+** | **+** | **+** |
| Probability (%) | **95** | **97** | **-** | **93** | **97** | **97** |

*Test reaction results appear as follows: negative (–) and positive (+).
